# Bee pollen in zebrafish diet affects intestinal microbiota composition and skin cutaneous melanoma development

**DOI:** 10.1038/s41598-022-14245-3

**Published:** 2022-06-15

**Authors:** Isabela M. Di Chiacchio, Elena Gómez-Abenza, Isadora M. Paiva, Danilo J. M. de Abreu, Juan Francisco Rodríguez-Vidal, Elisângela E. N. Carvalho, Stephan M. Carvalho, Luis David Solis-Murgas, Victoriano Mulero

**Affiliations:** 1 Programa de Pós-graduação em Ciências Veterinárias-FZMV, Universidade Federal de Lavras, UFLA, 3037, Lavras, MG 37200-900 Brasil; 2 Departamento de Biología Celular e Histología, Facultad de Biología, Universidad de Murcia. IMIB-Arrixaca. CIBERER, 30100 Murcia, Spain; 3 Laboratório de Genética Animal e Humana, Departamento de Genética, Ecologia e Evolução, Universidade Federal de Minas Gerais, UFMG, Belo Horizonte, MG 31270-901 Brasil; 4 Programa de Pós-graduação em Microbiologia Agrícola-ICN, Universidade Federal de Lavras, UFLA, 3037, Lavras, MG 37200-900 Brazil; 5 Departamento de Ciência dos Alimentos-ESAL, Universidade Federal de Lavras, UFLA, 3037, Lavras, MG 37200-900 Brasil; 6 Programa de Pós-graduação em Entomologia-ESAL, Universidade Federal de Lavras, UFLA, 3037, Lavras, MG 37200-900 Brasil

**Keywords:** Cancer, Immunology, Molecular biology

## Abstract

Bee pollen is recommended as dietary supplement due to immunostimulating functions including antioxidant, anti-inflammatory and anti-carcinogenic properties. Nevertheless, the effectiveness of such properties is still not well understood. As diet can be associated with animal performance, microbiota modulation and potentially factor for cancer, this study aimed to analyze if bee pollen could influence growth, gut microbial and skin cutaneous melanoma development in zebrafish. Control diets based on commercial flakes and *Artemia* were compared with the same diet supplemented with bee pollen. Fish weight gain, increased length, intestinal bacteria metagenomics analysis, serum amyloid A gene expression and cutaneous melanoma transplantation assays were performed. Bee pollen affected microbiota composition and melanoma development. Differential abundance revealed higher abundance in the control group for *Aeromonadaceae* family, *Aeromonas* and *Pseudomonas* genus, *A. sobria*, *A. schubertii*, *A. jandaei* and *P. alcaligenes* species compared with pollen diet group. Pollen group presented higher abundance for *Chromobacterium* genus and for *Gemmobacter aquaticus*, *Flavobacterium succinicans* and *Bifidobacterium breve* compared with control group. Unexpectedly, fish fed with bee pollen showed higher tumor growth rate and larger tumor size than control group. This is the first study to report intestinal microbial changes and no protective cancer properties after bee pollen administration.

## Introduction

Bee pollen is a natural food produced by bees to serve as a nutrient source for the colony development and maintenance. This product is particularly appreciated by consumers and used for therapeutic purposes due to its rich composition^1^. In bee pollen, approximately 250 different substances can be found^2^, amongst them nutrients as carbohydrates, proteins, vitamins, minerals, and fatty acids as well as secondary metabolites as phenolic compounds. Thus, many biological properties are attributed to it, such as antioxidant, antibacterial, antifungal, anti-inflammatory, antiallergic, hepatoprotective, and antitumor potential^3–7^. These pollen properties can vary depending on the origin and region of the plant, which directly affects its composition^8^.

Bee pollen in animal’s diet has been described especially related to the improvement in growth performance and immune status^9–13^. Besides, it is assumed that feed additives can alter intestinal microbiota, which in turn, interacts with the general host health, particularly affecting digestion, nutrients assimilation and modulation of the immune system^14^. The intestinal microbiota can influence the development and function of immune cells, such myeloid lineages as neutrophils through immune effectors. Serum amyloid A (Saa), one of the most highly induced transcripts in digestive tissues following microbiota colonization, serves as a systemic signal to neutrophils to restrict aberrant activation, decreasing inflammatory tone and bacterial killing potential while simultaneously enhancing their ability to migrate to wounds^15,16^.

To the best of our knowledge, the direct influence of dietary bee pollen through changes in the microbiota has still been little studied^17–20^. The detailed study of the intestinal microbiota composition and its metabolic functions allows determining which microorganisms make it possible to keep the intestine healthy and which changes can lead to pathologies development^21^. Intestinal microbiota generally maintains a constant relative pattern and altered bacterial abundance has been associated with complex diseases^22,23^. Dysbiosis of intestinal microbiota can be associated not only with intestinal but also with extra-intestinal diseases such as metabolic disorders^24^. The identification of diet–microbiome associations may be particularly relevant for studying the downstream effects of diet on long latency chronic diseases such as cancer^25^. In this context, increasing evidence also indicates a fundamental role of the microbiota in carcinogenesis^26–28^.

Several researches in cancer reveal that inflammation can play a key role from initiation of the transformed phenotype to metastatic spread. Chronic inflammation is considered one of the factors that most contribute to tumor appearance and progression^29,30^. In addition, the use of anti-inflammatory agents is shown to reduce tumor formation^30^ and natural products are also being used for cancer prevention or therapy and as adjuvants to conventional therapies^31–33^. Bee pollen has been described with both anti-inflammatory and anti-carcinogenic properties^1,3,34–38^, but many studies are still based on in vitro experiments.

Skin cutaneous melanoma (SKCM) is a dangerous type of skin cancer because of its potential for early metastasis and high mortality rate^39^. Nowadays, there is an increasing number of cases worldwide, many of them attributed to non-healing chronic wounds, scars and ulcers^40^. Recently it is discussed that factors beyond tumor genomics, including host habits such as diet and consequently their gastrointestinal microbiome, also influence cancer development and therapeutic responses^41–44^. Diet may be ubiquitous and one potentially modifiable risk factors for cancer, but despite the large global evidence base, the divergence in results are disappointingly common in this field^25^. Also, a recent study indicated that a favorable gut microbiome (high diversity and abundance of some specific bacteria) may modulate responses to immunotherapy in melanoma patients, enhancing systemic and antitumor immune responses in the periphery and in the tumor microenvironment^45^.

Given the unique advantages of the zebrafish model for molecular genetic analysis and in vivo imaging, together with the diverse set of research tools currently available, we believe it is a favorable model for study new therapeutic agents and mechanisms by which feed influences host. To date, there is no concrete and in-depth evidence on bee pollen prebiotic and antitumor effect. The present study aimed investigate if bee pollen addition in diet could influence zebrafish parameters. Fish diets based on commercial flakes and live food *Artemia* were offered as control and compared with the same diet supplemented with bee pollen and after diet administration period, fish weight gain, increased length, intestinal bacteria metagenomics analysis, serum amyloid A gene expression and skin cutaneous melanoma development after allotransplantation assays were performed.

## Methods

### Ethics statements

The experiments performed comply with the ARRIVE guidelines, with the Guidellines of the European Union Council (Directive 2010/63/EU) and the Spanish RD 53/2013. Experiments and procedures were performed as approved by the Consejería de Agua, Agricultura, Ganadería y Pesca de la CARM (authorization number #A13180602) and the Ethical Research in Animal Use Committee (CEUA) of Federal University of Lavras (approval number #001/18).

### Zebrafish husbandry

Zebrafish (*Danio rerio* H. Cypriniformes, Cyprinidae) were obtained from the Zebrafish International Resource Center (ZIRC, Oregon, USA) and mated, staged, raised and processed as described in the zebrafish handbook^46^. Zebrafish transgenic fish were held at our facilities following standard husbandry practices. Animals were maintained in a 12 h light/dark cycle at 28 °C. *Tg(kita:GalTA4,UAS:mCherry)*^*hzm1*^ zebrafish were crossed with *Tg(UAS:eGFP-H-RAS_G12V)*^*io6*^ line^47^ to express oncogenic human HRAS_G12 V driven by the melanocyte cell-specific promoter *kita*. The transparent *roy*^*a9/a9*^; *nacre*^*w2/w2*^ (casper)^48^ of 4–8 month old were previously described.

### Experimental diets

The experimental design was divided into groups with 2 different types of diets, according to Table 1. Adult zebrafish were fed three times a day, divided into 3 aquariums per treatment. All groups received different diets at the same time (9:00 a.m., 12:00 p.m. and 3:00 p.m.). Tropical Fish Flakes (Prodac, Italy) were employed, which are routinely utilized in the laboratory and are highly recommended for the species. The bee pollen samples (produced by *Apis mellifera*) were obtained from apiary located in the city of Neópolis (10° 19′ 13″ S, 36° 34′ 41″ W), Sergipe State, Brazil) and at our laboratory were crushed and sieved (0.5 mm) to enable ingestion by the animals. The feed amount offered by individual was 3% of body weight—BW (flakes and bee pollen), per meal, and the number of brine shrimp *Artemia* nauplii (48 h nauplii) offered was 2000 per individual per day (food protocol already established in the laboratory). To ensure intake of the desired amount of pollen by all animals and to avoid food selection, pollen was offered separately from flakes and brine shrimp, in a single meal.

**Table 1:** Distribution of diets by experimental group.

Groups	1st meal	2nd meal	3rd meal
	9:00 h	12:00 h	15:00 h
1—Control	Flakes^a^	Flakes	Artemia^b^
2—Pollen	Flakes	Bee pollen^c^	Artemia

^a^Tropical Fish Flakes (Prodac, Italy): cereals, fish and fish products, soy, yeast, crustaceans, algae, aloe vera and mineral and vitamin mixture. ^b^Brine shrimp (Inve Aquaculture, Thailand). ^c^Neópolis, SE, Brazil.

For brine shrimp (Inve Aquaculture, Thailand) hatching, cysts were subjected to the following protocol: incubation for 48 h with filtered marine water, at 28 °C under intense aeration until nauplii hatching followed by collection of nauplii after washing in fresh water immediately before being offered to the animals.

Composition and proximate analysis of fish flakes and brine shrimp offered in the animals' basal diet are described in Table 2 (data obtained from the manufacturers). Information not provided by the manufacturer was found in the literature^49^.

**Table 2:** Composition and proximate analysis of fish flakes and brine shrimp (data provided by manufacturers) offered in the animals' basal diet.

Composition	Proximate analysis (%) Artêmia	
	Flakes flakes	Brine shrimp
Crude protein	44.9	55.0
Ether extract	4.47	13.0
Ash	4.35	5.5
Fiber	2.14	6.8^a^
Moisture	7.73	68^a^
Carbohydrates	NI	13.22^a^

Values expressed for each 100 g of dry matter. *NI* no information available. ^a^Average values by Rizk et al.^49^. Nutritional additives: vitamin A, 41.200 I.U./kg; vitamin D3, 3.000 I.U./kg; vitamin E, 297 mg/kg; vitamin C, 180 mg/kg.

Composition, proximate analysis and antioxidant capacity of bee pollen (triplicate determination) are also listed in Table 3, according to analyzes performed at the Department of Food Sciences, University of Lavras, Brazil.

**Table 3:** Composition, proximate analysis and antioxidant capacity of bee pollen.

Composition	Mean ± SD
Moisture	14.56 ± 0.05
Crude protein	17.57 ± 0.04
Ether extract	5.14 ± 0.12
Carbohydrates	60.38 ± 0.16
Total sugar	25.59 ± 0.26
Reducing sugars	24.78 ± 0.12
Sacarose	0.82 ± 0.14
Ashes^a^	3.02 ± 0.04
**Antioxidant capacity**	
Phenolic content (mgGAE/g)	19.15 ± 4.13
ABTS (μmol trolox/mg)	3955.30 ± 27.05

Data are represented as mean ± standard deviation (n = 3). Values expressed for each 100 g of dry matter. All values are in accordance with the Ministry of Agriculture, Cattle and Supplying (MAPA) normative instruction 3 (annex V) which addresses requirements for bee products commercialization in Brazil. ^a^Mineral analysis: N, 34.8 g/kg; P, 6.57 g/kg; K, 6.73 g/kg; Ca, 5.92 g/kg; Mg, 2.18 g/kg; S, 2.22 g/kg; B, 6.07 mg/kg; Cu, 11.69 mg/kg; Mn, 222.24 mg/kg; Zn, 64.40 mg/kg; Fe, 106.07.

### Increased length and weight gain

After 60 days of feeding with control and pollen-based diets, fish from each treatment were anesthetized in buffered 0.16 mg/mL trincaine (Sigma Aldrich) for growth parameters measurements. The growth parameters were determined according to following formula:

$${\text{Mean}}\,{\text{weight}}\,{\text{gain }}\left( {{\text{WG}}} \right) \, = {\text{ Mean}}\,{\text{final}}\,{\text{weight }} - {\text{ Mean}}\,{\text{initial}}\,{\text{weight,}}$$

$${\text{Increased}}\,{\text{length }}\left( {{\text{IL}}} \right) \, = {\text{ Mean}}\,{\text{final}}\,{\text{length }}{-}{\text{ Mean}}\,{\text{initial}}\,{\text{length}}{.}$$

### Sample collection and genomic DNA extraction

Fish from each treatment (n = 3) were transferred into well cleaned separate tanks and after 24 h of starvation period were anesthetized and euthanized according the European Union Council and IUAC protocol (tricaine overdose: 1.2 mg/mL; Sigma Aldrich). Then, their intestines were removed, quickly frozen in liquid nitrogen inside 1.5 mL tubes containing 500 µL of RNAlater™ stabilization solution (Invitrogen, Thermo Fisher) and subsequently preserved at − 80 °C until DNA extraction and samples preparation. The bacterial genomic DNA was extracted using a PureFood GMO and Authentication kit (Maxwell® RSC, Promega, USA) following the manufacture’s protocol.

### Intestinal microbiota assessment through metagenomics analysis

The intestinal microbial composition of animals (n = 3) fed with 2 different diets was determined by sequencing 16S rRNA gene. The “Ion 16S Metagenomics Kit" (Ion Torrent) used includes primers to amplify variable regions V2, V4 and V8 in a single tube with ~ 250 base pair (bp), ~ 288 bp and ~ 295 amplicons bp, respectively, and in a second tube, a multiplex PCR reaction directed to variable regions V3, V6, V7 and V9 with ~ 215 bp, ~ 260 bp and ~ 209 bp, respectively. The primers are designed to capture > 80% sequences found in Greengenes database with 100% identity (BARB et al. 2016). For 16S rRNA PCR amplification, maximum DNA amount (6 µL) was used following conditions indicated in the protocol (25 cycles). PCR products were verified by 2% agarose gel electrophoresis, purified with AMPure XP Beads (Beckman Coulter), quantified with “Qubit dsDNA HS Assay” kit (Invitrogen) using 50 ng of total amplicons to generate "Ion" libraries Plus Fragment Library Kit "(Ion Torrent). The model was prepared using the Ion OneTouch™ 2 system and the “Ion PGM™ Template Hi-Q view OT2 400” kit (Ion Torrent). The sequencing was performed using the "Ion PGM™ Sequencing Hi-Q view 400" kit (Ion Torrent) in the Ion PGM™ system. Samples with microbial identification were analyzed at family, genus and species level.

### Analysis of proinflammatory gene transcript levels by RT-qPCR

Animals from each treatment had their total RNA extracted from zebrafish intestines (n = 5) using TRIzol reagent (Invitrogen), and then purified with Mini Kit total RNA purification system (Ambion) and treated with DNase I, amplification grade (1 U/μg RNA; Thermo Fisher Scientific). The SuperScript IV RNase Reverse Transcriptase (Thermo Fisher Scientific) was used to synthesize first-strand cDNA with oligo(dT)_18_ primer from 1 μg of total RNA at 50 °C for 50 min. Real-time PCR was performed with a QuantStudio 5 (Thermo Fisher Scientific) using SYBR Green PCR Core Reagents (Applied Biosystems). Reaction mixtures were incubated for 10 min at 95 °C, followed by 40 cycles of 15 s at 95 °C, 1 min at 60 °C, and finally 15 s at 95 °C, 1 min 60 °C and 15 s at 95 °C. For each mRNA quantified, gene transcription was normalized in relation to the ribosomal protein S11 (*rps11*) housekeeping gene by Pfaffl method^50^. The primers sequences were: Serum amyloid A (S*aa*) F: 5′-CGCAGAGGCAATTCAGAT-3′ and R: 5′-CAGGCCTTTAAGTCTGTATTTGTTG-3′; Interleukin 1 beta (*Il1b*) F: 5′-GCCTGTGTGTTTGGGAATCT-3′ and R: 5′-TGATAAACCAACCGGGACAT-3′; Tumor Necrosis Factor Alpha (*Tnfa*) F: 5′-GCGCTTTTCTGAATCCACG-3′ and R: 5′-TGCCAGTCTGTCTCCTTCT-3′; Leukotriene A4 Hydrolase (*Lta4h*) F: 5′-AATCTCATGAGCAATGACAC-3′ and R: 5′-CATTTGTCACTCCAACTGTG-3′; C-Reactive Protein (*Crp*) F: 5′-GCTCTCTGTGACATTAGAGGCTA-3′ and R: 5′-CTGTTGTCAGTAGCGGTGTTG-3′. Each PCR was performed with triplicate samples.

### SKCM transplant

The zebrafish Casper line (n = 22) fed with different diets (120 days) were used as recipients for melanoma transplantation (SKCM). Zebrafish *kita:Gal4*; *eGFP-HRAS-G12V*, which express human oncogenic HRAS in melanocytes and spontaneously develop SKCM, were used as tumor donors (n = 2) for allotransplantation assays. All the next procedures were developed according to a previous study^51^. Briefly, primary melanoma tumors were excised from adult zebrafish once they had reached between 3 and 5 mm in diameter and right after the procedure individuals were euthanized with an overdose of tricaine (1.2 mg/mL). The tumor was excised with scalpel and razor blade, placed in 2 mL of disaggregation media, composed by DMEM/F12 (Life Technologies), penicillin/streptomycin (Life Technologies) and 0.075 mg/mL of Liberase (Roche). After manually disaggregation with a clean razor blade and incubation at room temperature for 30 min, 5 mL of wash media, composed by DMEM/F12, penicillin/streptomycin, and 15% heat-inactivated fetal bovine serum (FBS, Life Technologies), was added to the tumor slurry and manually disaggregated. Next, the tumor cells suspensions were passed through a 40 μm filter (BD) into a clean 50 mL tube. An additional 5 mL of wash media was added to the initial tumor slurry that was filtered again. This procedure was repeated twice. Cell numbers were calculated with a hemocytometer and the tubes of resuspended cells were centrifuged at 800*g* for 5 min at 4 °C. The pellet of tumor cells was resuspended in the appropriate volume of PBS containing 5% FBS and kept on ice prior to transplantation^52^.

After fasting 48 h, adult zebrafish used as transplant recipients, were immunosuppressed to prevent rejection of the donor material. Thus, the recipients were anesthetized, as previously described, and treated with 30 Gy (Gy) of split dose sub-lethal X-irradiation (YXLON SMART 200E, 200 kV, 4.5 mA) two days before the transplantation. Then the immunosuppressed fish were maintained in carefully clean fish water with conditions preventing any infections onset and, consequently, preventing recipients’ deaths. The animals were anesthetized with a double protocol, according to studies using longer anesthetic protocols (up to 40 min)^52^. Briefly, anesthesia was first induced by tricaine (Sigma-Aldrich) and then the fish were transferred to tricaine/isoflurane solution (dilution in ethanol, 1:9). Anesthetized fish (10–20 per tumor) were placed dorsal side up on a damp sponge and injections were performed using a 10 μL beveled, 26S-guaged Hamilton syringe, needle positioned midline and ahead to the dorsal fin. Three-hundred thousand cells resuspended in PBS were injected into the dorsal subcutaneous cavity. The syringe was washed in 70% ethanol and rinsed with PBS between uses.

Following transplantation, fish were placed into recovery tanks and weekly evaluated for melanoma formation. SKCM cells used in our study show enhanced aggressiveness in adult zebrafish allotransplantation model and fish recipients transplanted can develop tumors with a significant high growth rate^51^. Thus, photographs from adult transplantation assays were obtained at 1, 2, 3 and 4 weeks post injection (wpi). Zebrafish were anesthetized, placed in a dish of fish water, and photographed using a mounted camera (Nikon D3100 with a Nikon AF-S Micro Lens). The pigmented tumor size was represented by the number of pigmented pixels (Adobe Photoshop CS5).

### Statistical analysis

All data were analyzed for normality by the Shapiro–Wilk test. Data (except metagenomics) were analyzed using *GraphPad Prism* 7.01 by one or two-way analysis of variance (ANOVA) and a Tukey or Sidak post-test for multiple comparisons evidencing differences between groups. The survival curves were analyzed using the log-rank (Mantel-Cox) test. Statistical significance was defined as *p ≤ 0.05; **p ≤ 0.01; ***p ≤ 0.001.

Data from IonReporter program were analyzed using R Core Team 2019 to find statistically significant differences (differential abundance) in taxa composition between different diets. Thus, the abundance data were normalized by dividing the abundance value by the total number of sample readings and multiplied by 100,000 to guarantee values greater than 1 or 0 in the absence of a taxon in the sample. Finally, data were converted to phyloseq^53^ to generate the diversity graphs and converted to DESeq2^54^ to perform the differential abundance statistical test. DESeq performs a differential analysis based on the negative binomial distribution.

## Results

### Bee pollen inclusion in diet presented similar growth parameters as control

**Figure 1:**
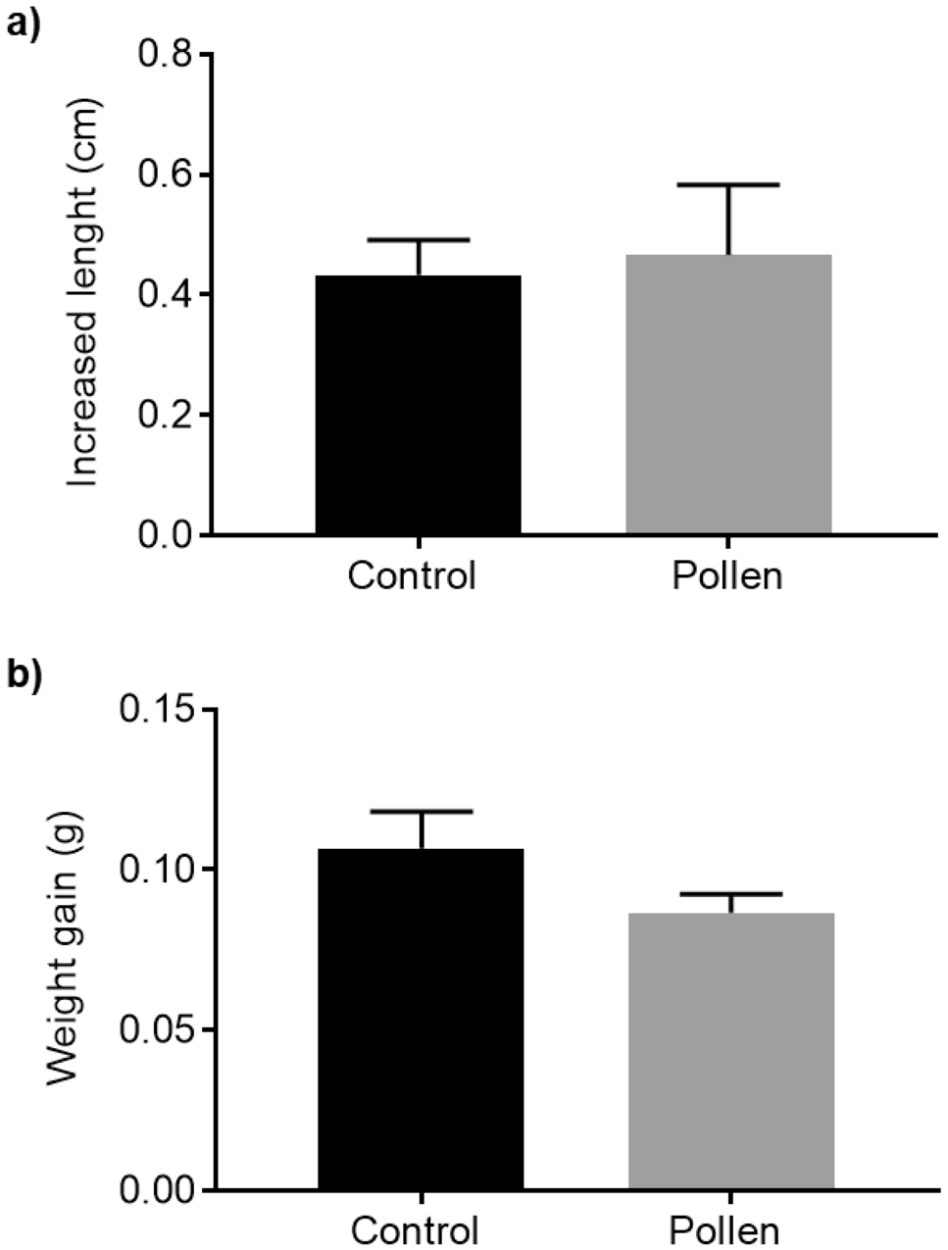
Growth parameters of adult zebrafish after feeding with control diet (black bar) vs. pollen diet (gray bar). (**a**) Increased length (cm). (**b**) Mean weight gain (g). p > 0.05 according to unpaired Student t test. The data are shown as mean + SEM (n = 24).

Zebrafish growth parameters after the feed regime period (60 days) is shown in Fig. 1. No significant differences (p > 0.05) were found between control diet and pollen supplemented diet for both measurements: increased length (Fig. 1a) and mean weight gain (Fig. 1b). Fish from the group fed with control diet had a mean growth of 0.43 ± 0.06 cm and 0.10 ± 0.012 g and fish from the group fed with pollen diet achieved a mean growth of 0.47 ± 0.12 cm and 0.09 ± 0.005 g.

### Bee pollen diet induced gut microbial changes

**Figure 2:**
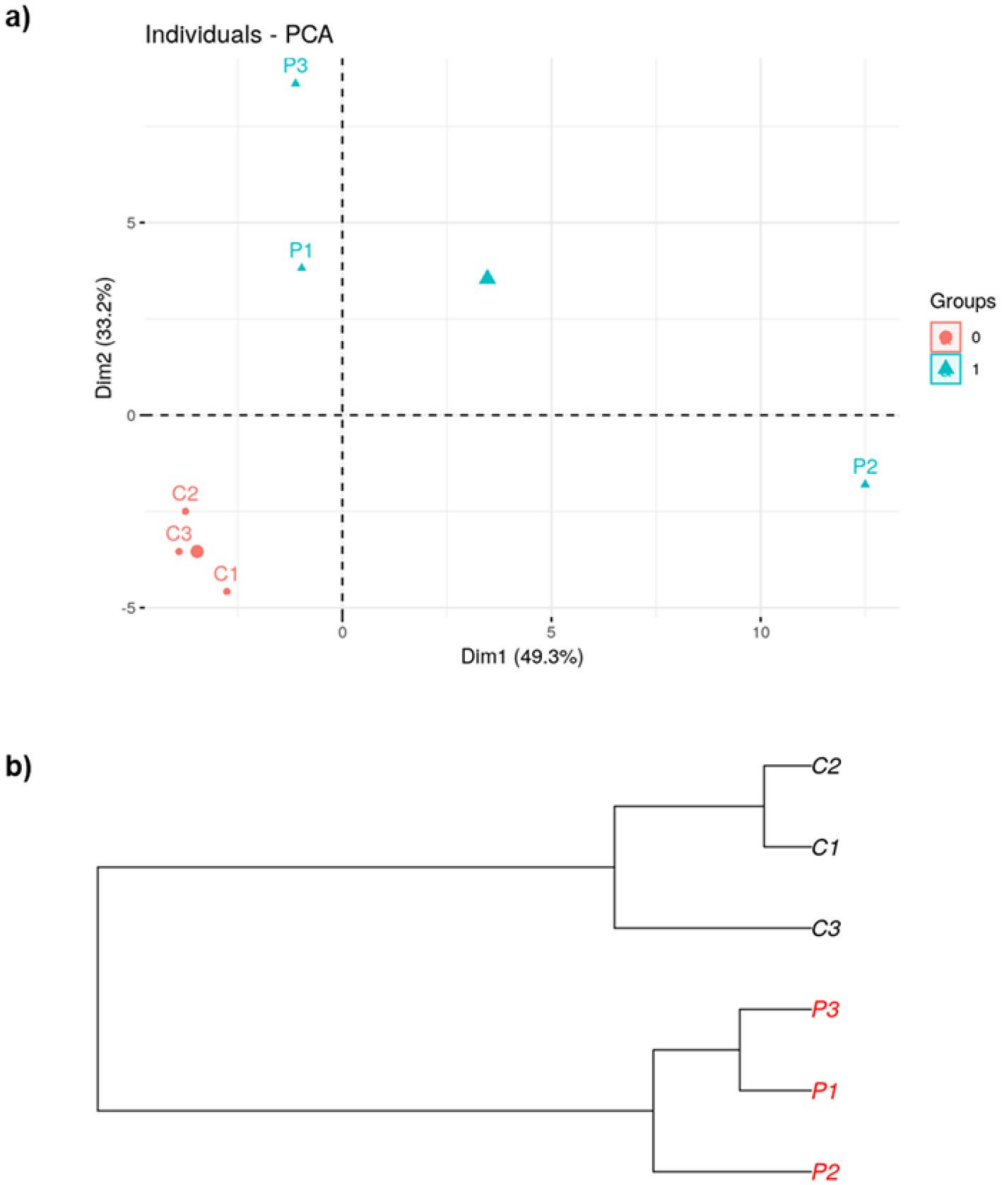
Relationship between the composition of the gut bacterial communities in zebrafish fed with control diet (C_1–3_) and pollen supplemented diet (P_1–3_). (**a**) Principal Component Analysis (PCA) plot. (**b**) Dendrogram. Generated by R Core Team 2019.

**Figure 3:**
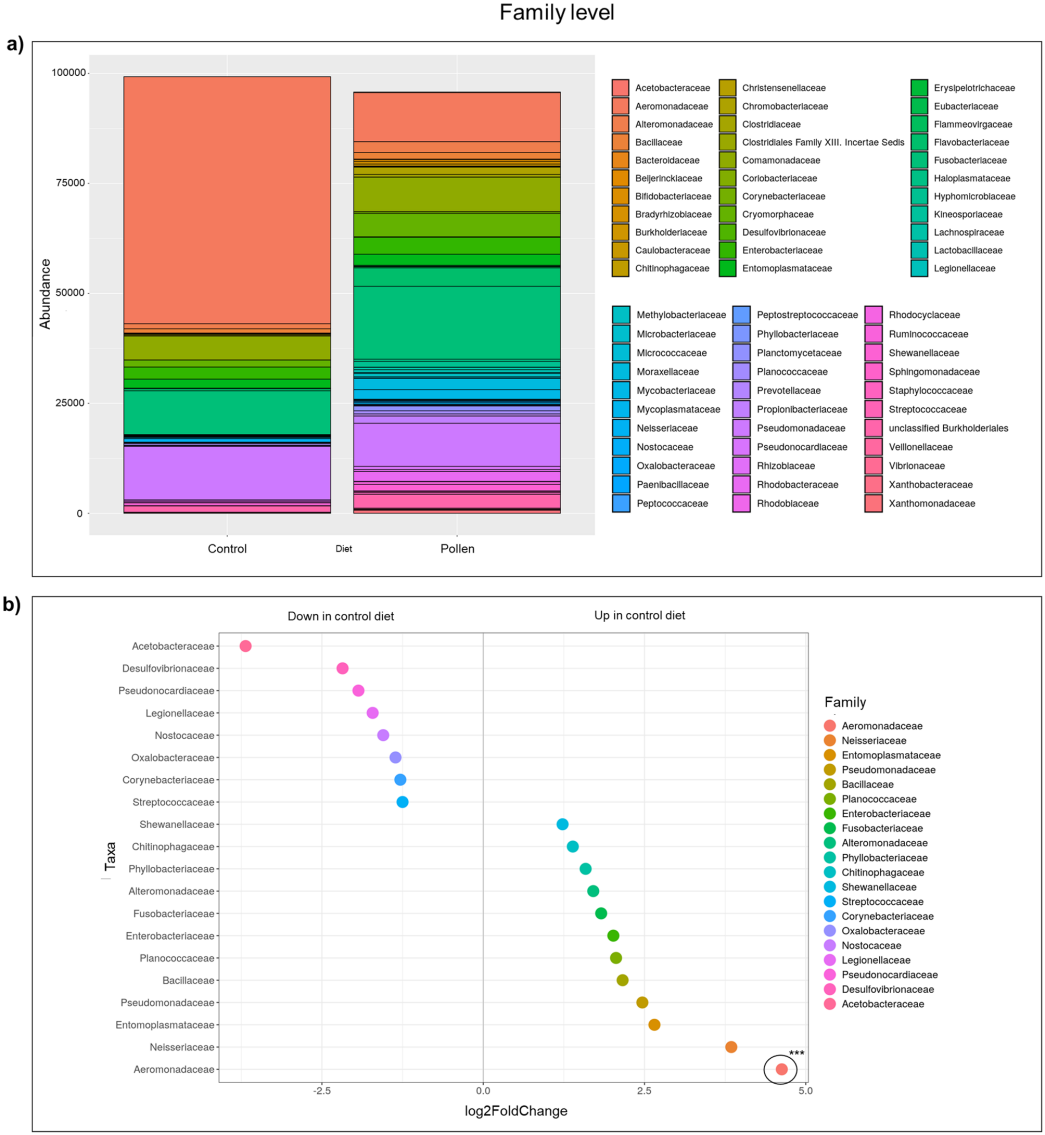
Bacterial communities at family level. (**a**) Stacked column bar graph showing the distribution and abundances of bacteria in zebrafish fed with control diet and pollen supplemented diet. (**b**) Dot plot graph showing significantly different abundant OTUs (***p < 0.001), where OTUs are grouped by color family along the y-axis. The x-axis indicates the log2 fold-change in control diet compared to pollen diet.

**Figure 4:**
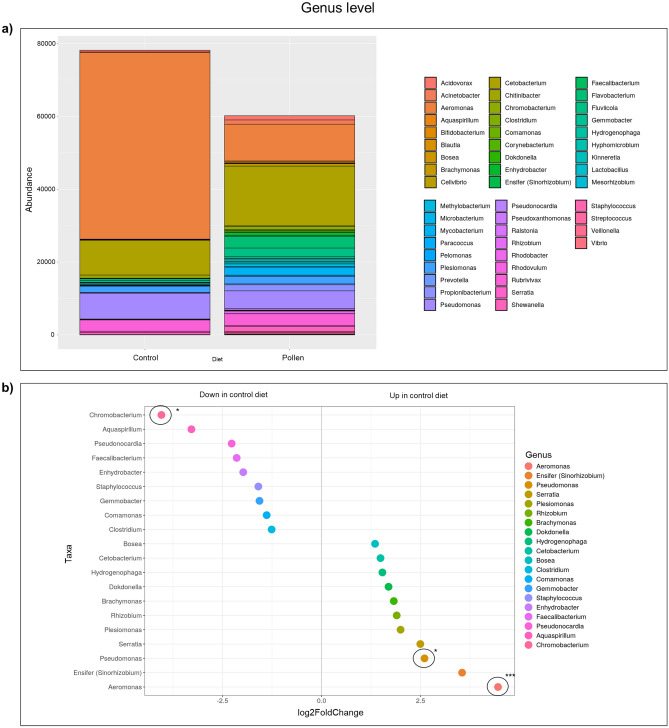
Bacterial communities at genus level. (**a**) Stacked column bar graph showing the distribution and abundances of bacteria in zebrafish fed with control diet and pollen supplemented diet. (**b**) Dot plot graph showing significantly different abundant OTUs (*p < 0.05; ***p < 0.001), where OTUs are grouped by color along the y-axis. The x-axis indicates the log2 fold-change in control diet compared to pollen diet.

**Figure 5:**
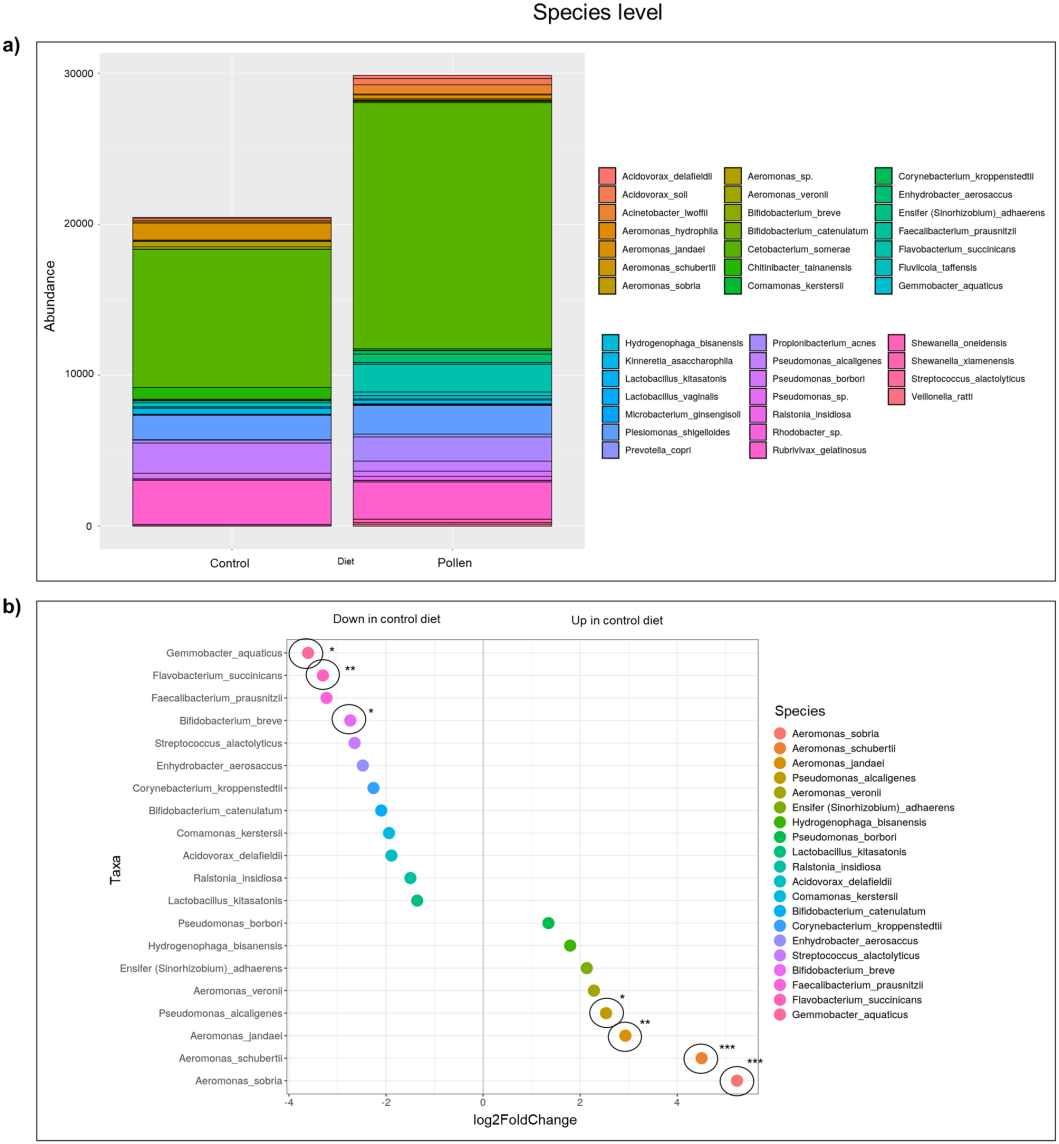
Bacterial communities at species level. (**a**) Stacked column bar graph showing the distribution and abundances of bacteria in zebrafish gut fed with control diet and pollen supplemented diet. (**b**) Dot plot graph showing significantly different abundant OTUs (*p < 0.05; **p < 0.01; ***p < 0.001), where OTUs are grouped by color along the y-axis. The x-axis indicates the log2 fold-change in control diet compared to pollen diet.

Metagenomics analyses from zebrafish gut microbiome after control and pollen diets are shown in Figs. 2, 3, 4 and 5. The PCA plot (Fig. 2a) and dendrogram (Fig. 2b) showed a closely related microbial community within each sample. The dendrogram analysis also supported the PCA plot clustering by showing the robustness of the differences between control and pollen supplemented diet samples.

Abundance data (quantitative values obtained from operational taxonomic unit, OTU) for each diet group were compared. OTUs were taxonomically grouped and differential abundance analyzed at the family, genus and species levels revealed that the microbiome of pollen supplemented group showed significantly altered abundance compared to the control diet fish. Stacked column bar graph illustrate the distribution and abundances of bacterial communities in zebrafish samples (control diet—C_1–3;_ pollen supplemented diet—P_1–3_). Each bacterial taxon was represented by different color (Figs. 3, 4 and 5).

At the family level, control diet group presented significantly higher abundance (p < 0.001) for *Aeromonadaceae* compared to pollen diet group (Fig. 3). At the genus level, control diet group presented significantly higher abundance for *Aeromonas* (p < 0.001) and *Pseudomonas* (p < 0.05) compared with pollen diet group, while pollen diet group presented higher abundance for *Chromobacterium* (p < 0.05) compared with control fish (Fig. 4). At the species level, control diet group presented significantly higher abundance (p < 0.001) for *Aeromonas sobria* (p < 0.001), *A. schubertii* (p < 0.001), *A. jandaei* (p < 0.01) and *Pseudomonas alcaligenes* (p < 0.05) compared to pollen diet group, while pollen group presented higher abundance for *Gemmobacter aquaticus* (p < 0.05), *Flavobacterium succinicans* (p < 0.01) and *Bifidobacterium breve* (p < 0.05) compared to control group (Fig. 5).

### Similar transcript levels of genes encoding proinflammatory mediators for bee pollen and control fed fish

**Figure 6:**
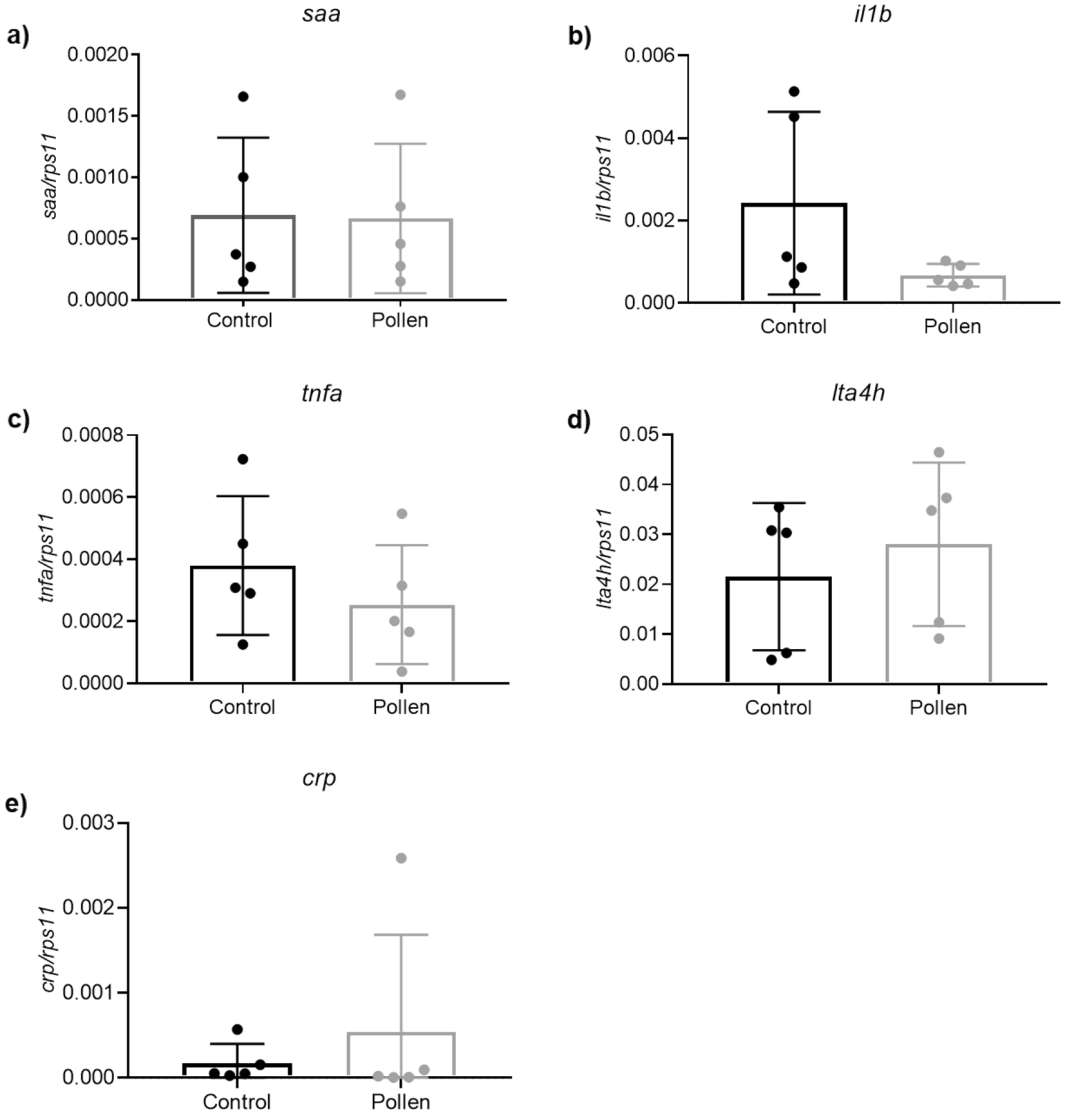
The transcript levels of genes encoding proinflammatory mediators in zebrafish intestines after diet treatments. (**a**) *saa*. (**b**) *il1b*. (**c**) *tnfa*. (**d**) *lta4h*. (**e**) *crp*. p > 0.05 according to unpaired Student t test. The data are shown as mean + SEM (n = 5).

As bee pollen has been shown to have anti-inflammatory properties^1,6^ and we found that it altered the microbiota of zebrafish, we analyzed the transcript levels of several genes encoding key proinflammatory mediators, including Saa, Il1b, Tnfa, Lta4h and Crp in zebrafish intestines (Fig. 6). Our results revealed similar transcript levels of these genes in the intestine of zebrafish fed with bee pollen and their control counterparts.

### Bee pollen diet induced higher tumor growth after SKCM transplant

**Figure 7:**
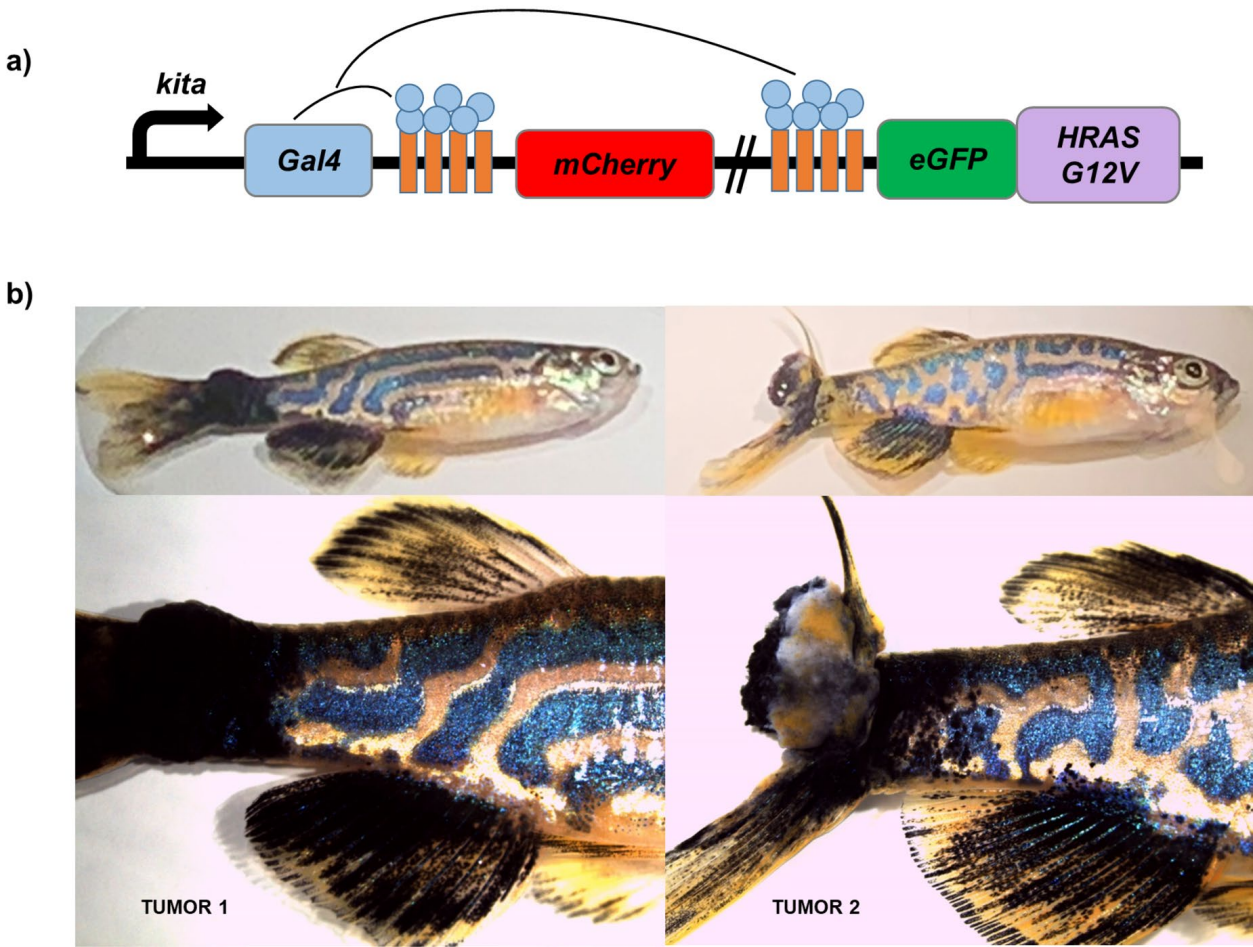
Animals used as tumor donors for transplantation. (**a**) Schematic diagram of SKCM model line in zebrafish. *Tg (kita:GalTA4,UAS:mCherry)*^*hzm1*^ zebrafish was crossed with *Tg (UAS:eGFP-H-RAS_G12V)*^*io6*^ line to express oncogenic human HRAS_G12V driven by the melanocyte cell-specific promoter *kita*. (**b**) Representative images of *kita:Gal4*;*eGFP-HRAS-G12V* whole fish and nodular tail tumor (1 and 2) used in our study (biopsied and disaggregated for posterior allotransplantation).

**Figure 8:**
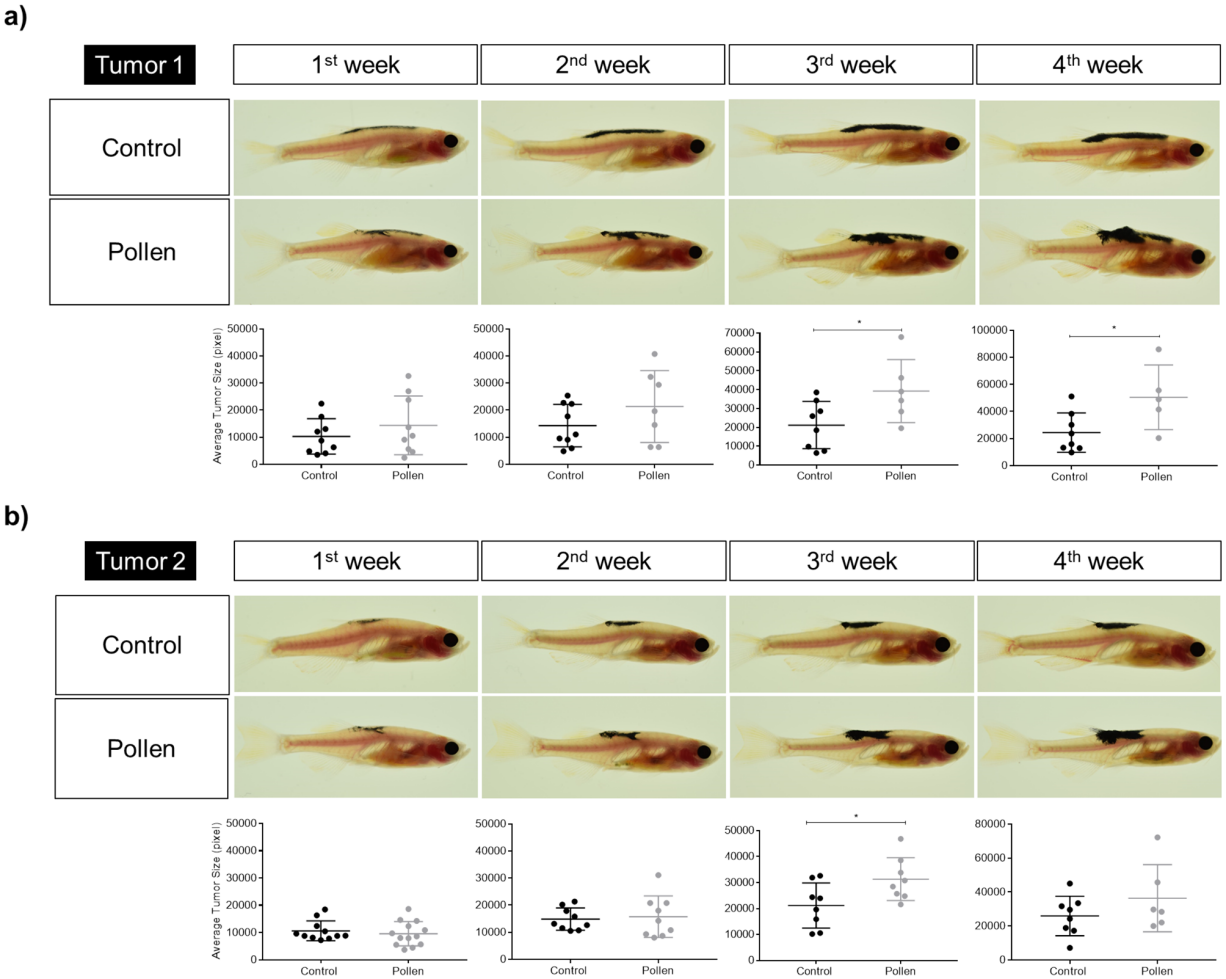
Tumors representative images and average tumor size (pixels) from 1 to 4 weeks’ post-transplant. (**a**) Tumor 1. (**b**) Tumor 2. Each dot corresponds to a recipient-transplanted fish and the mean ± SEM is also shown. *p < 0.05 according to unpaired Student t test.

**Figure 9:**
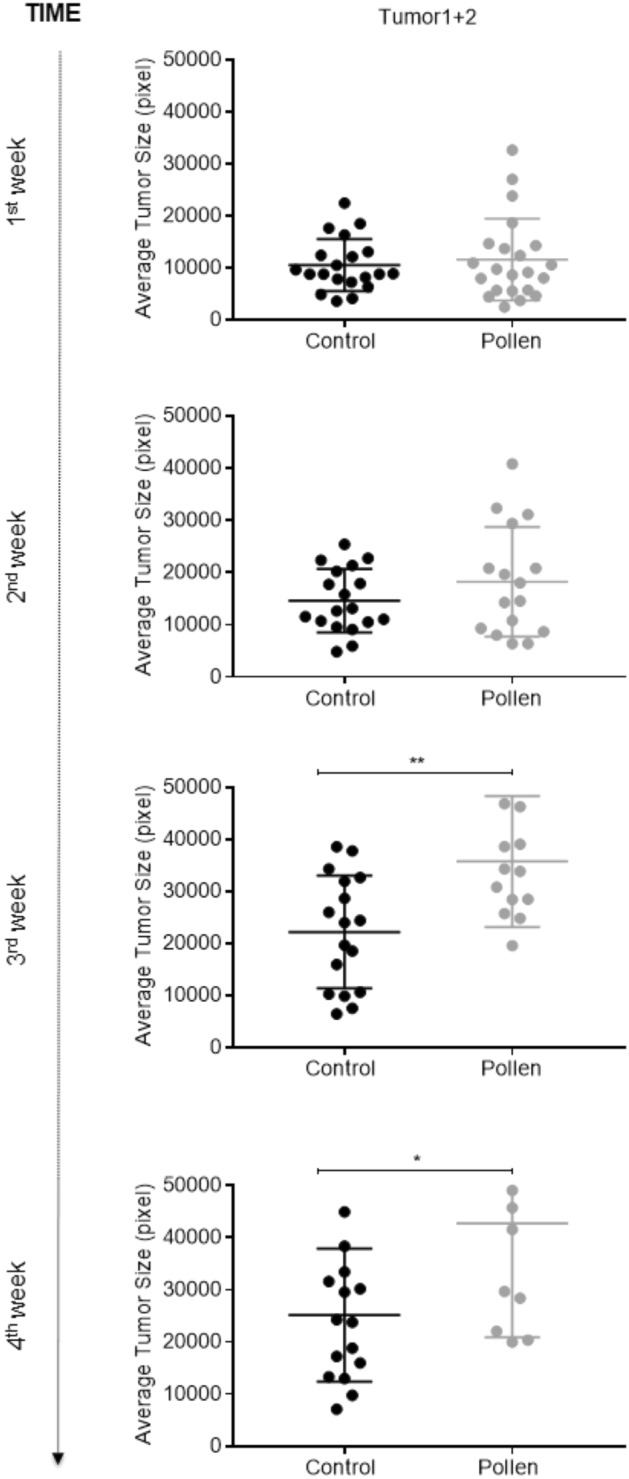
Average tumor size. Average tumor (1 + 2) size (pixels) from 1 to 4 weeks’ post-transplant. Each dot corresponds to a recipient-transplanted fish and the mean ± SEM is also shown. *p < 0.05, **p < 0.01 according to unpaired Student t test.

**Figure 10:**
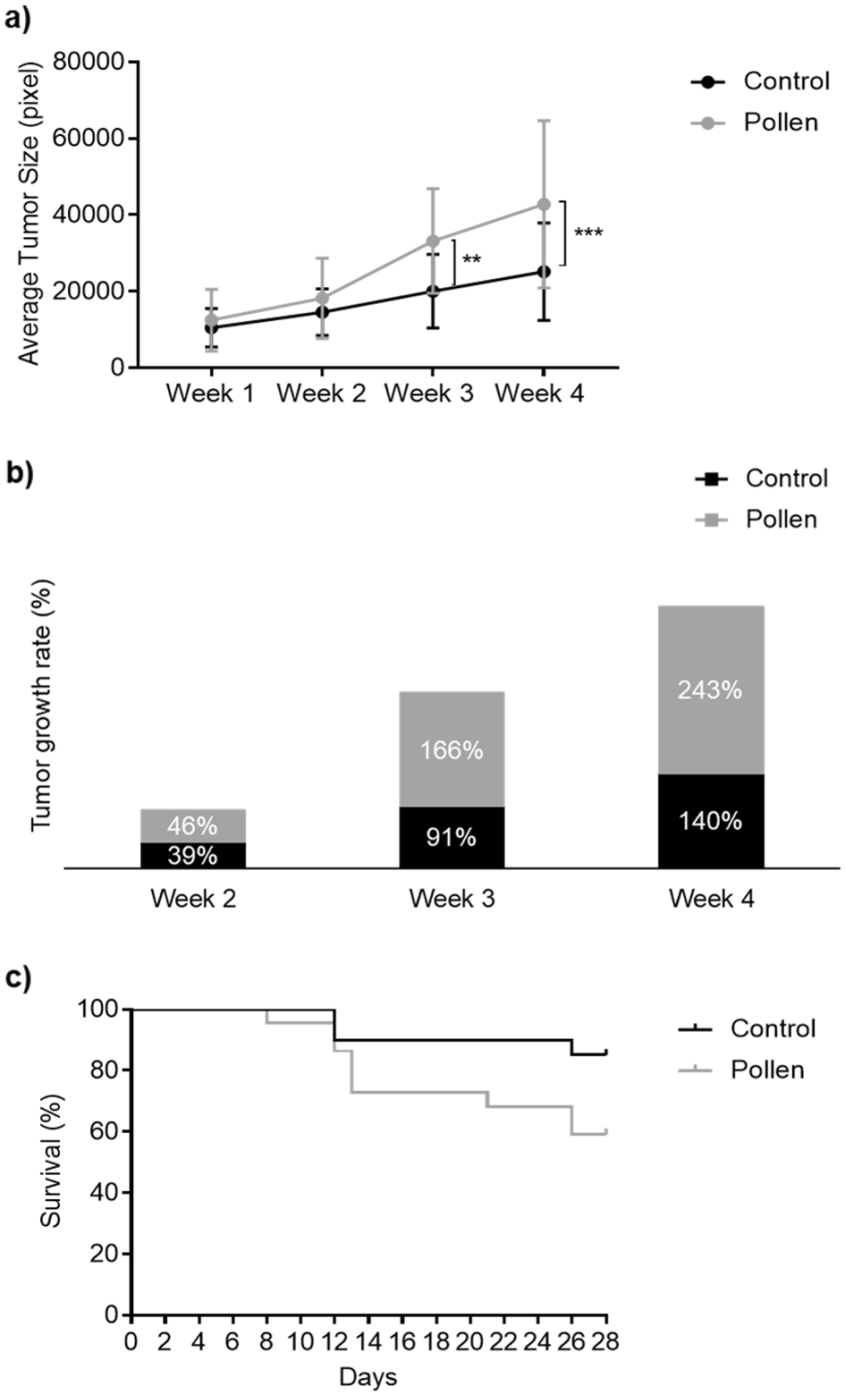
Adult casper zebrafish fed with control diet (black color) vs. pollen diet (gray color) over 4 weeks after melanoma allotransplant. (**a**) Average tumor (1 + 2) size (pixels). **p < 0.01; ***p < 0.001 according to ANOVA and Sidak’s Multiple Comparison Test. (**b**) Tumor growth rate (%). (**c**) Survival curve (%). Kaplan–Meier Gehan–Breslow–Wilcoxon and nonparametric Log-rank Test.

Zebrafish SKCM allotransplantation process and tumor cell proliferation and dissemination in vivo assays are described by Figs. 7, 8, 9 and 10. Figure 7a shows a schematic diagram of *kita:Gal4;eGFP-HRAS-G12V* and representative images of whole fish and nodular tail tumor (1 and 2) used as melanoma donors in our study are shown in Fig. 7b.

Analyzing separately tumor 1 and 2 transplantation for different diet groups, pigmented tumors engrafted were scored during 4 weeks for tumor size and in the first and second weeks of analysis there was find no significantly differences (p > 0.05) between the treatments (Fig. 8a,b). At the third week of analysis, zebrafish fed with bee pollen developed tumors with significant (p < 0.05) larger tumor size (mean of 35,225 pixels for tumor 1 and 31,348 pixels for tumor 2) compared with zebrafish fed with control diet (mean of 19,083 pixels for tumor 1 and 23,020 pixels for tumor 2). At the fourth week, bee pollen group developed tumors with significant (p < 0.05) larger size compared to control only for tumor 1 (mean of 50,511 pixels for pollen group and 24,434 pixels for control group), while tumor 2 presented no difference (p > 0.05) between treatments (mean of 36,326 pixels for bee pollen group and 25,871 pixels for control group). Representative images of Tumor 1 and 2 engraftment and tumor size average from week 1 to 4 post-transplantation are presented in Fig. 8a,b.

Figure 9 shows tumor 1 and 2 analyzed together and both showed a similar pattern. At the first and second weeks, no differences (p > 0.05) were observed between the 2 treatments. From the third week of analysis, zebrafish fed with bee pollen developed tumors with larger (p < 0.01) tumor size (mean of 33,157 pixels in the third week, 42,774 pixels in the fourth week) compared to no pollen-fed fish (mean of 20,045 pixels at third week, 25,152 pixels at fourth week). Melanoma recipients fed with pollen and transplanted with SKCMs (tumor 1 + 2) also presented tumors with higher (p < 0.01) growth rate (166% at the third week, 243% at the fourth week) than those recipients fed with control diet (91% in the third week, 140% in the fourth week) (Fig. 10a,b). In relation to recipient survival curve, no significant differences (p > 0.05) were observed between diet groups during the 4 weeks analyzed (Fig. 10c).

## Discussion

We here describe effects of bee pollen administration that have never been reported or that contradict many works in the literature on other species.

Our results do not show any significant effect of dietary bee pollen in growth performance in zebrafish. Nevertheless, supplementing diets with bee pollen has been reported to improved growth parameters in other species, as calves^55^, rabbits^10,56^, and also in fish Nile tilapia *Oreochromis niloticus*^9,11^. Additionally, studies with rats suggested increased intestinal absorptive capacity and nutrient usability in bee pollen fed animals^13,57^. Improvements in growth characteristics (length and weight gain) of bee pollen fed animals may be attributed to its components, like vitamins, minerals and enzymes or coenzymes, which may enhance digestion and assimilation of nutrients^58^. However, we believe that responses to pollen feeding can vary according to the species studied, the control-based diet, the concentration offered and the nutritional composition of each pollen.

The addition of pollen in the diet has also demonstrated effects on rat’s intestine mucosal surface, causing a slight increase in epithelial layer of the small intestine and significantly increased the epithelium volume and decreased the connective tissue volume^12^. These results may be related to positive changes found in other studies for growth parameters, but they can also indicate important changes in the animals' digestive tract and consequences in other structures, such as the microbiota. Thus, we hypothesized bee pollen could cause changes in zebrafish intestinal microorganisms.

Gut microbiota may vary according to the intestine anatomical regions, which changes in terms of physiology, pH and oxygen tension, digesta flow rates, substrate availability, and host secretions^59,60^. Generally, fecal samples are accepted for microbiome investigations, but tissue biopsy containing multiple regions of the gastrointestinal tract has demonstrated to achieve a more comprehensive and appropriate representation of the microbial communities contributing to gut tissue health^61–63^. In accordance, we have sampled the entire zebrafish gut tissue in our study. To the best of our knowledge, this is the first study reporting the effects of bee pollen feeding on zebrafish intestinal microbiota.

Phenolic compounds, especially flavonoids, present in the wall of pollen grains are the main substances related to biological and therapeutic activities^1^. These substances were shown to have an important influence on some specific bacteria in bee’s intestinal microbiota, as *Bifidobacterium asteroides,* increasing the production of several metabolites (juvenile hormone derivatives and prostaglandins) that have key functions in immunity and physiology of these animals^64^. There is few information about bee pollen influencing the intestinal microbiome in other species but, interestingly, *Lactobacillus* and *Bifidobacterium*, widespread used as probiotics for humans and animals, have been isolated from bee pollen samples^65–67^.

In our study, we found that bee pollen affected intestinal microbiota composition with differential abundance at family, genus and species levels. The regulation of multiple host metabolic pathways, as homeostasis and immunostasis, is performed by gut microbiota^68^. Nonetheless, little is known about the function of individual gut bacterium in zebrafish. Different zebrafish facilities share what is called “core gut microbiota”, which the dominant phyla are Proteobacteria (mostly the genera *Aeromonas* and *Shewanella*), Fusubacteria or Firmicutes (*Bacilli* class), Actinobacteria and Bacteroidetes^69^. However, the composition of the resident gut microbes is also modulated by diet, which plays a vital role in many bacteria’s diversity and/or abundance^26^. Although microbiota composition is relatively stable, permanent changes (dysbiosis) may occur due to dietary and environmental alterations^70^.

In the present study, pollen diet group presented significantly lower abundance at family level for *Aeromonadaceae* and at genus level for *Aeromonas* and *Pseudomonas. Aeromonas* and *Pseudomonas* spp. are genus commonly found in aquatic environments^71,72^. Some studies described *Aeromonas* spp. as the only group of bacteria that are present throughout the zebrafish life cycle, suggesting the existence of this bacteria in the core microbiota with important colonization resistance functionality. They seem to play important roles in immune defense, gut cell growth, and inducing the transcription of important genes^73–75^. It is known that the genus *Aeromonas* sp. also secretes an immunomodulatory protein called AimA that prevents the recruitment of excessive intestinal neutrophils^76^. In addition, both genus can be of great economical and medical importance, since members of this genus are distributed in freshwater and in association with aquatic animals are sometimes known to cause a diverse spectrum of diseases^77^.

Notwithstanding, at species level, we have identified *A. sobria*, *A. schubertii, A. jandaei*, and *P. alcaligenes* with significantly lower abundance at pollen diet group. Although they can be isolated from fish intestinal tracts, these *Aeromonas* species have also been described as animals and human’s pathogens, associated with gastrointestinal problems, wound infections, septicemia, enterotoxin production and represent an important economic problem in aquaculture^78–81^. *P. alcaligenes* has been also isolated as pathogen in fish causing hemorrhagic disease^82^. Studies are still necessary to elucidate the role of each individual bacterium in the microbiota, as well as the effects of the complex interaction between different microorganisms to achieve a beneficial balance.

Pollen diet group presented significantly higher abundance at genus level for *Chromobacterium*. Species of the genus *Chromobacterium* have been described with probiotic effects. For example, *Chromobacterium violaceum*, which produce violacein, a violet pigment that possesses functions such as antibacterial, antiviral, antifungal, and antioxidant activities, was shown to have an impact in the mammalian gut microbiome^83^. Changes in rat’s microbial diversity were found after orally violacein administration, modulating specially components of Firmicutes and Actinobacteria phyla. In fact, studies have demonstrated violacein immunomodulatory potential, and yet antitumor activity^84^. Also, *Chromobacterium aquaticum* administered as a probiotic, after isolated from lake water samples, could modulate zebrafish immunity against *A. hydrophila* and *S. iniae*, as well as enhance its nutrient metabolism and growth performance^85^. The probiotic produced extracellular enzymes and a substance similar to bacteriocin, which improved bactericidal activity against pathogens.

At species level, higher abundance for *Gemmobacter aquaticus, Flavobacterium succinicans* and *Bifidobacterium breve* were found in our study for bee pollen group. Although little is known about *G. aquaticus* and *F. succinicans*, *Bifidobacterium breve* has been described as effective probiotic bacteria. For example, it is widely used by humans, especially in pediatric areas, since it has antimicrobial activity against human pathogens and immuno-stimulating abilities^86,87^. Also, an interesting study showed that oral administration of commensal *Bifidobacterium* as probiotic promoted antitumor immunity (improving the function of dendritic cells and consequently increased infiltration of effector T-tumor cells) and controlled the growth of melanoma in mice, indicating that the composition of commensal microbial can also influence spontaneous anti-tumor immunity, as well as responses to immunotherapy. Oral administration of the probiotic improved tumor control to the same degree as specific antibody therapy for the tumor programmed cell death protein 1 ligand (PD-L1) and in a treatment with both combined, tumor outgrowth were almost abolished^88^. In mice, *Bifidobacterium breve* was shown to effectively induce the Regenerating islet-derived III (REGIII; one class of antimicrobials protein expressed in the intestine) production via the MyD88-Ticam1 pathway, demonstrating that this probiotic may enhance the mucosal barrier and protect the host from infection and inflammation^89^.

The transcript levels of genes encoding key proinflammatory mediators in the intestines were performed in our study to see if dietary pollen directly or indirectly through the alteration of the microbiota could results in intestinal inflammation. Our results suggest that dietary bee pollen does not results in intestinal inflammation, since the transcript levels of all genes analyzed were unaltered. The unaltered expression of *saa* gene is of great relevance, since serum amyloid A is a conserved secreted protein produced in the intestine and liver and with described effects on immune cells as neutrophils. Notably, it has been shown that the microbiota is able to induce the gene encoding Saa expression in the zebrafish intestine and this Saa produced in response to microbiota serves as a systemic signal to neutrophils to enhance their ability to migrate to wounds^15^. Also, in our previously work we found that offspring of zebrafish fed with bee pollen supplemented diets showed higher neutrophil migration to wounds^90^. If microorganism’s diversity can lead to varied levels of Saa protein, this factor could facilitate specific effects on host innate immune system^15^. Some authors described some bacteria, such as *Pseudomonas aeruginosa*, *Aeromonas hydrophila* and *Escherichia coli*, to strongly induce Saa transcriptions, while others such as *Shewanella* sp. and *Staphylococcus* sp. failed to modulate the same gene^73^. It is assumed that a complex interaction of different microorganisms in the digestive tract stimulates the more potent expression of proteins and immune markers compared to individual strains, indicating that may be necessary a combination of specific microorganisms to alter the mRNA levels of these genes. Whatever the outcome, our results suggest that dietary bee pollen does not increase melanoma growth by promoting intestinal inflammation.

A unique optimal gut microbiota composition does not exist since it is different for everyone. However, a healthy host–microorganism balance must be respected in order to optimally perform metabolic and immune functions and prevent disease development^24^. There is a close mutualistic relationship between gut microbiota variations and diseases, including extra-intestinal diseases such as metabolic disorders^24^. With this in mind, we have decided to study if pollen supplementation in diet, together with the changes in the intestinal microbiota found, could influence cancer development. Thus, SKCM allotransplantation assay was performed in Casper zebrafish to directly visualize tumor cell proliferation and dissemination in vivo over time.

Bee pollen has been linked to anti-carcinogenic properties^1,34,35^ but there is still no full evidence for this attribution. Studies have shown bee pollen with greater or lesser antimutagenic properties in different types of cancer^3,36–38^. These activities may be derived from its antioxidant properties (mainly suppression of oxygen reactive species formation)^1^, its ability to induce apoptosis and stimulate secretion of tumor necrosis factor-alpha^2,91^, cytotoxic activity on cells^6^, and by simply enhancing and strengthening the immune system^92^. Thus, in accordance with results obtained mostly in cell cultures, it has been suggested that bee pollen extracts containing different types of compounds, especially phenolic acids and flavonoids (e.g. kaempferol, apigenin), help to control cell growth^1^. Epidemiological studies about a diet rich in natural polyphenols show that many of this compounds could lower the risk of certain cancers by mechanisms of action mainly associated with cell survival, proliferation, differentiation, migration, angiogenesis, hormone activities, detoxification enzymes and immune responses^93^. Nonetheless, the difficult in assessing intake of dietary polyphenols through bee pollen ingestion, the diversity of polyphenols in each sample and their different bioavailability might contribute to inconsistent results. Besides, the anticancer effects may vary with cancer types, cell lines and doses. Literature data suggests that natural polyphenols, could reduce the incidence of different types of cancers including prostate, colon, breast, lung, bladder, pancreatic and skin cancer^94^.

Nowadays, skin cancers are attributed to chronically injured, non-healing wounds, scars or ulcers^40^. Some studies suggest that bee pollen may also affect the wound healing process of burn wounds^95^. In this context, we hypothesized whether it could have a beneficial effect on melanoma development. In our study, bee pollen supplementation in zebrafish diet had no protective properties against SKCM. Pre-clinical studies suggest that many compounds derived from natural products have potent activity against cancer cells or xenotransplanted tumors and that they can prevent the carcinogenesis or metastasis of existing tumors^32^. Instead, we observed a stimulating growth effect. A study proposed that patients with a favorable gut microbiome enhance systemic and antitumor immune responses and, by contrast, patients with an unfavorable gut microbiome have impaired systemic and antitumor immune responses^45^. Regarding our results, it is possible that changes in the microbiota found in pollen group may have interfered with tumor progression; or even the pollen composition, with a high level of carbohydrates and sugars, could interfere negatively in the response to tumor development. Some studies propose that higher levels of blood glucose and insulin are cancer risk factors. Insulin has been shown to stimulate cell division, supporting the growth and spread of cancer cells and making them more difficult to eliminate^96–98^. In addition, higher levels of insulin and blood glucose can lead to the growth of abnormal cells and possibly contribute to cancer^96^. The bee pollen used in our study was composed by 60% of carbohydrates and high content of total sugar, which could have affected both microbiota composition and response to cancer. Cancer cells usually have high levels of glucose uptake and metabolism, which plays an important role in tumor growth. Some studies have demonstrated that natural polyphenols could be used for the prevention and treatment of cancer by inhibiting glucose cellular uptake in addition to antioxidant and anti-inflammation effects^93^. A very recent publication about the effect of pollen supplementation in mice fed a high-fat/high-sucrose diet showed a decreased fasting blood glucose, increased glucose-stimulated insulin secretion, and resulted in changes of the gut microbiota^18^. Correlations between genus abundances and metabolic changes in response to supplementation also indicated that the gut microbiota contributed to the positive effects of pollen ingestion on fasting glucose^18^. In our study, we provided a high concentration of pollen in the diet, and this may also have resulted in higher ingestion of polyphenols and have potentially positive effects. However, the high amount of some macronutrients, such as carbohydrates and sugars, in the bee pollen used in our study can diverge effects on blood sugar, insulin metabolism and changes in the gut microbiota. It would be interesting for future studies to analyze blood glucose levels and evaluate this correlation with tumor development and changes in the microbiota after bee pollen administration. Bee pollen is not a natural food for fish and the effects of its inclusion in the diet are not yet known. Future studies analyzing different doses of bee pollen administration in fish would help to clarify this issue. In addition, would be also very interesting analyze other types of cancer after pollen ingestion, since the diet can affect the tumor microenvironment in different pathways and dietary factors could influence cancers along the digestive tract differently than other types of cancer^99^.

Due to its variable composition, the effects caused by bee pollen ingestion cannot be simply generalized and its use should be prudent. There is a large number of different substances, which can interfere individually and even with complex interactions between them. Studies with bee substances is challenging and deserves greater attention in future studies. In conclusion, bee pollen as dietary supplement did not affect zebrafish weight gain, increased length or serum amyloid A gene expression, but changed intestinal microbiota composition and had a stimulant effect on SKCM development.

## Data Availability

The data presented in this study are available on request from the corresponding author.

## References

[1] Denisow B, Denisow-Pietrzyk M (2016). Biological and therapeutic properties of bee pollen: A review. J. Sci. Food Agric..

[2] Komosinska-Vassev K, Olczyk P, Kaźmierczak J, Mencner L, Olczyk K (2015). Bee pollen: Chemical composition and therapeutic application. Evid. Based Complement. Altern. Med..

[3] Abdella EM, Tohamy A, Ahmad RR (2009). Antimutagenic activity of Egyptian propolis and bee pollen water extracts against cisplatin-induced chromosomal abnormalities in bone marrow cells of mice. Int. J. Cancer Manag..

[4] Nogueira C, Iglesias A, Feás X, Estevinho LM (2012). Commercial bee pollen with different geographical origins: A comprehensive approach. Int. J. Mol. Sci..

[5] Fatrcová-Šramková K (2013). Antioxidant and antimicrobial properties of monofloral bee pollen. J. Environ. Sci. Health Part B Pestic Food Contam. Agric. Wastes.

[6] Pascoal A, Rodrigues S, Teixeira A, Feás X, Estevinho LM (2014). Biological activities of commercial bee pollens: Antimicrobial, antimutagenic, antioxidant and anti-inflammatory. Food Chem. Toxicol..

[7] De-Melo, A. A. M. & de Almeida-Muradian, L. B. Chemical composition of bee pollen. In *Bee Products—Chemical and Biological Properties* 221–259 (Springer International Publishing, 2017). 10.1007/978-3-319-59689-1_11.

[8] De-Melo AAM (2018). Phenolic profile by HPLC-MS, biological potential, and nutritional value of a promising food: Monofloral bee pollen. J. Food Biochem..

[9] Abbass AA, El-Asely AM, Kandiel MMM (2012). Effects of dietary propolis and pollen on growth performance, fecundity and some hematological parameters of *Oreochromis niloticus*. Turk. J. Fish. Aquat. Sci..

[10] Attia YA, Al-Hanoun A, Tag El-Din AE, Bovera F, Shewika YE (2011). Effect of bee pollen levels on productive, reproductive and blood traits of NZW rabbits. J. Anim. Physiol. Anim. Nutr. (Berl).

[11] El-Asely AM, Abbass AA, Austin B (2014). Honey bee pollen improves growth, immunity and protection of *Nile tilapia* (*Oreochromis niloticus*) against infection with *Aeromonas hydrophila*. Fish Shellfish Immunol..

[12] Hajkova Z, Toman R, Galik B (2014). The effect of bee pollen consumption on functional morphology of small intestine of rats. Conference MendelNet.

[13] Wang J, Li S, Wang Q, Xin B, Wang H (2007). Trophic effect of bee pollen on small intestine in broiler chickens. J. Med. Food.

[14] López Nadal A (2020). Feed, microbiota, and gut immunity: Using the Zebrafish model to understand fish health. Front. Immunol..

[15] Murdoch CC (2019). Intestinal serum amyloid a suppresses systemic neutrophil activation and bactericidal activity in response to microbiota colonization. PLoS Pathog..

[16] Sack GH (2018). Serum amyloid A—A review. Mol. Med..

[17] Cheng N, Chen S, Liu X, Zhao H, Cao W (2019). Impact of schisandrachinensis bee pollen on nonalcoholic fatty liver disease and gut microbiota in highfat diet induced obese mice. Nutrients.

[18] Rebelo KS (2022). Pot-pollen supplementation reduces fasting glucose and modulates the gut microbiota in high-fat/high-sucrose fed C57BL/6 mice. Food Funct..

[19] Xu Y (2021). Impact of *Camellia japonica* bee pollen polyphenols on hyperuricemia and gut microbiota in potassium oxonate-induced mice. Nutrients.

[20] Chen S, Zhao H, Cheng N, Cao W (2019). Rape bee pollen alleviates dextran sulfate sodium (DSS)-induced colitis by neutralizing IL-1β and regulating the gut microbiota in mice. Food Res. Int..

[21] Preidis GA, Versalovic J (2009). Targeting the human microbiome with antibiotics, probiotics, and prebiotics: Gastroenterology enters the metagenomics era. Gastroenterology.

[22] Shreiner AB, Kao JY, Young VB (2015). The gut microbiome in health and in disease. Curr. Opin. Gastroenterol..

[23] Durack J, Lynch SV (2019). The gut microbiome: Relationships with disease and opportunities for therapy. J. Exp. Med..

[24] Rinninella E (2019). What is the healthy gut microbiota composition? A changing ecosystem across age, environment, diet, and diseases. Microorganisms.

[25] Murphy R (2020). An integrative approach to assessing diet–cancer relationships. Metabolites.

[26] Mandal RS, Saha S, Das S (2015). Metagenomic surveys of gut microbiota. Genom. Proteomics Bioinform..

[27] Raza MH (2019). Microbiota in cancer development and treatment. J. Cancer Res. Clin. Oncol..

[28] Schwabe RF, Jobin C (2013). The microbiome and cancer. Nat. Rev. Cancer.

[29] Coussens LM, Werb Z (2002). Inflammation and cancer. Nature.

[30] Singh N (2019). Inflammation and cancer. Ann. Afr. Med..

[31] Rayburn ER, Ezell SJ, Zhang R (2009). Anti-inflammatory agents for cancer therapy. Mol. Cell. Pharmacol..

[32] Strimpakos AS, Sharma RA (2008). Curcumin: Preventive and therapeutic properties in laboratory studies and clinical trials. Antioxid. Redox Signal..

[33] Aggarwal BB, Shishodia S (2006). Molecular targets of dietary agents for prevention and therapy of cancer. Biochem. Pharmacol..

[34] Kieliszek M (2018). Pollen and bee bread as new health-oriented products: A review. Trends Food Sci. Technol..

[35] Li QQ (2018). Nutrient-rich bee pollen: A treasure trove of active natural metabolites. J. Funct. Foods.

[36] Furusawa E, Chou SC, Hirazumi A, Melera A (1995). Antitumour potential of pollen extract on lewis lung carcinoma implanted intraperitoneally in syngeneic mice. Phyther. Res..

[37] Wan Omar WA, Azhar NA, Harif Fadzilah N, Nik Mohamed Kamal NNS (2016). Bee pollen extract of Malaysian stingless bee enhances the effect of cisplatin on breast cancer cell lines. Asian Pac. J. Trop. Biomed..

[38] Uçar M (2016). Effect of Turkish pollen and propolis extracts on caspase-3 activity in myeloid cancer cell lines. Trop. J. Pharm. Res..

[39] Siegel RL, Miller KD, Jemal A (2020). Cancer statistics, 2020. CA. Cancer J. Clin..

[40] Tang L, Wang K (2016). Chronic inflammation in skin malignancies. J. Mol. Signal..

[41] Garrett WS (2015). Cancer and the microbiota. Science (80-)..

[42] Elinav E, Garrett WS, Trinchieri G, Wargo J (2019). The cancer microbiome. Nat. Rev. Cancer.

[43] Segre JA (2015). Microbial growth dynamics and human disease: Examining microbial genome replication in tissues may reflect health status. Science (80-)..

[44] Drewes JL, Housseau F, Sears CL (2016). Sporadic colorectal cancer: Microbial contributors to disease prevention, development and therapy. Br. J. Cancer.

[45] Gopalakrishnan V (2018). Gut microbiome modulates response to anti-PD-1 immunotherapy in melanoma patients. Science (80-)..

[46] Westerfield, M. *ZFIN: The Zebrafish Book. A Guide for the Laboratory Use of Zebrafish.* (University of Oregon Press, 2007).

[47] Santoriello C (2010). Kita driven expression of oncogenic HRAS leads to early onset and highly penetrant melanoma in zebrafish. PLoS ONE.

[48] White RM (2008). Transparent adult Zebrafish as a tool for in vivo transplantation analysis. Cell Stem Cell.

[49] Rizk E-ST, Shoukr FA, El-Gamal MM, Abdel-Razek FA, Mona MM (2018). An attempt to improve the proximate composition of local Artemia strain (Wadi El Natrun, Egypt). J. Basic Appl. Zool..

[50] Pfaffl MW (2001). A new mathematical model for relative quantification in real-time RT-PCR. Nucleic Acids Res..

[51] Gómez-Abenza E (2019). SPINT1 regulates the aggressiveness of skin cutaneous melanoma and its crosstalk with tumor immune microenvironment. bioRxiv.

[52] Dang M, Henderson RE, Garraway LA, Zon LI (2016). Long-term drug administration in the adult Zebrafish using oral gavage for cancer preclinical studies. DMM Dis. Model. Mech..

[53] McMurdie PJ, Holmes S (2013). phyloseq: An R package for reproducible interactive analysis and graphics of microbiome census data. PLoS One.

[54] Love MI, Huber W, Anders S (2014). Moderated estimation of fold change and dispersion for RNA-seq data with DESeq2. Genome Biol..

[55] Tu Y, Zhang GF, Deng KD, Zhang NF, Diao QY (2015). Effects of supplementary bee pollen and its polysaccharides on nutrient digestibility and serum biochemical parameters in Holstein calves. Anim. Prod. Sci..

[56] Zeedan K, El-Neney BAM, Aboughaba AAAA, El-Kholy K (2017). Effect of bee pollen at different levels as natural additives on immunity and productive performance in rabbit males. Egypt. Poult. Sci..

[57] Hajková Z (2013). The effect of pollen on the structure of the small intestine in rats after an experimental addition in diet. Sci. Pap. Anim. Sci. Biotechnol. Lucr. Stiint. Zooteh. si Biotehnol..

[58] Xu X, Sun L, Dong J, Zhang H (2009). Breaking the cells of rape bee pollen and consecutive extraction of functional oil with supercritical carbon dioxide. Innov. Food Sci. Emerg. Technol..

[59] Flint HJ, Scott KP, Louis P, Duncan SH (2012). The role of the gut microbiota in nutrition and health. Nat. Rev. Gastroenterol. Hepatol..

[60] Valdes AM, Walter J, Segal E, Spector TD (2018). Role of the gut microbiota in nutrition and health. BMJ.

[61] Huse SM (2014). Comparison of brush and biopsy sampling methods of the ileal pouch for assessment of mucosa-associated microbiota of human subjects. Microbiome.

[62] Bashir M (2016). Effects of high doses of vitamin D3 on mucosa-associated gut microbiome vary between regions of the human gastrointestinal tract. Eur. J. Nutr..

[63] Koo H (2017). Metagenomics approach to the study of the gut microbiome structure and function in zebrafish *Danio rerio* fed with gluten formulated diet. J. Microbiol. Methods.

[64] Kešnerová L (2017). Disentangling metabolic functions of bacteria in the honey bee gut. PLoS Biol..

[65] Asama T (2015). *Lactobacillus kunkeei* YB38 from honeybee products enhances IgA production in healthy adults. J. Appl. Microbiol..

[66] Vásquez A, Olofsson TC (2009). The lactic acid bacteria involved in the production of bee pollen and bee bread. J. Apic. Res..

[67] Anderson KE (2013). Microbial ecology of the hive and pollination landscape: Bacterial associates from floral nectar, the alimentary tract and stored food of honey bees (*Apis mellifera*). PLoS One.

[68] Merrifield, D. L. & Rodiles, A. The fish microbiome and its interactions with mucosal tissues. In *Mucosal Health in Aquaculture* 273–295 (Elsevier Inc., 2015). 10.1016/B978-0-12-417186-2.00010-8

[69] Roeselers G (2011). Evidence for a core gut microbiota in the zebrafish. ISME J..

[70] Blumberg R, Powrie F (2012). Microbiota, disease, and back to health: A metastable journey. Sci. Transl. Med..

[71] Mena KD, Gerba CP (2009). Risk assessment of pseudomonas aeruginosa in water. Rev. Environ. Contam. Toxicol..

[72] Gonçalves Pessoa RB (2019). The genus Aeromonas: A general approach. Microb. Pathog..

[73] Rawls JF, Mahowald MA, Ley RE, Gordon JI (2006). Reciprocal gut microbiota transplants from zebrafish and mice to germ-free recipients reveal host habitat selection. Cell.

[74] Stephens WZ (2016). The composition of the zebrafish intestinal microbial community varies across development. ISME J..

[75] Burns AR, Guillemin K (2017). The scales of the zebrafish: Host–microbiota interactions from proteins to populations. Curr. Opin. Microbiol..

[76] Rolig AS (2018). A bacterial immunomodulatory protein with lipocalin-like domains facilitates host–bacteria mutualism in larval zebrafish. Elife.

[77] Sugita H, Tanaka K, Yoshinami M, Deguchi Y (1995). Distribution of Aeromonas species in the intestinal tracts of river fish. Appl. Environ. Microbiol..

[78] Igbinosa IH, Igumbor EU, Aghdasi F, Tom M, Okoh AI (2012). Emerging Aeromonas species infections and their significance in public health. Sci. World J..

[79] Liu JY, Li AH (2012). First case of *Aeromonas schubertii* infection in the freshwater cultured snakehead fish, *Ophiocephalus argus* (Cantor), China. J. Fish Dis..

[80] Beaz-Hidalgo R, Figueras MJ (2013). Aeromonas spp. whole genomes and virulence factors implicated in fish disease. J. Fish Dis..

[81] Yu J, Koo BH, Kim DH, Kim DW, Park SW (2015). Aeromonas sobria infection in farmed mud loach (*Misgurnus mizolepis*) in Korea, a bacteriological survey. Iran. J. Vet. Res..

[82] Xu J, Zeng X, Jiang N, Zhou Y, Zeng L (2015). *Pseudomonas alcaligenes* infection and mortality in cultured Chinese sturgeon, *Acipenser sinensis*. Aquaculture.

[83] Pauer H (2018). Impact of violacein from *Chromobacterium violaceum* on the mammalian gut microbiome. PLoS One.

[84] Durán N (2016). Advances in *Chromobacterium violaceum* and properties of violacein-its main secondary metabolite: A review. Biotechnol. Adv..

[85] Yi CC, Liu CH, Chuang KP, Chang YT, Hu SY (2019). A potential probiotic *Chromobacterium aquaticum* with bacteriocin-like activity enhances the expression of indicator genes associated with nutrient metabolism, growth performance and innate immunity against pathogen infections in zebrafish (*Danio rerio*). Fish Shellfish Immunol..

[86] Cionci NCB, Baffoni L, Gaggìa F, Di Gioia D (2018). Therapeutic microbiology: The role of bifidobacterium breve as food supplement for the prevention/treatment of paediatric diseases. Nutrients.

[87] Cukrowska B, Bierła JB, Zakrzewska M, Klukowski M, Maciorkowska E (2020). The relationship between the infant gut microbiota and allergy. The role of Bifidobacterium breve and prebiotic oligosaccharides in the activation of anti-allergic mechanisms in early life. Nutrients.

[88] Sivan A (2015). Commensal Bifidobacterium promotes antitumor immunity and facilitates anti-PD-L1 efficacy. Science (80-)..

[89] Natividad JMM (2013). Differential induction of antimicrobial REGIII by the intestinal microbiota and Bifidobacterium breve NCC2950. Appl. Environ. Microbiol..

[90] Di Chiacchio IM (2021). Bee pollen as a dietary supplement for fish: Effect on the reproductive performance of zebrafish and the immunological response of their offspring. Fish Shellfish Immunol..

[91] Wu YD, Lou YJ (2007). A steroid fraction of chloroform extract from bee pollen of *Brassica campestris* induces apoptosis in human prostate cancer PC-3 cells. Phyther. Res..

[92] Wang B (2013). Antitumor activity of bee pollen polysaccharides from *Rosa rugosa*. Mol. Med. Rep..

[93] Zhou Y (2016). Natural polyphenols for prevention and treatment of cancer. Nutrients.

[94] Briguglio G (2020). Polyphenols in cancer prevention: New insights (review). Int. J. Funct. Nutr..

[95] Olczyk P (2016). Bee pollen as a promising agent in the burn wounds treatment. Evid. Based Complement. Altern. Med..

[96] Paoli A, Rubini A, Volek JS, Grimaldi KA (2013). Beyond weight loss: A review of the therapeutic uses of very-low-carbohydrate (ketogenic) diets. Eur. J. Clin. Nutr..

[97] Rose DP, Vona-Davis L (2012). The cellular and molecular mechanisms by which insulin influences breast cancer risk and progression. Endocr. Relat. Cancer.

[98] Denley A (2007). Differential activation of insulin receptor substrates 1 and 2 by insulin-like growth factor-activated insulin receptors. Mol. Cell. Biol..

[99] Key TJ (2020). Diet, nutrition, and cancer risk: What do we know and what is the way forward?. BMJ.

